# Medical School Faculty Diversity and the Liaison Committee on Medical Education’s Diversity Standards

**DOI:** 10.1001/jamanetworkopen.2025.12096

**Published:** 2025-05-22

**Authors:** Mytien Nguyen, Tonya L. Fancher, Sarwat I. Chaudhry, Alan Dardik, Laura Castillo-Page, Gbenga Ogedegbe, Paris Butler, Mayur M. Desai, Shruthi Venkataraman, Olivia Marie Campa, Amy Sage, Dowin Boatright

**Affiliations:** 1 Department of Immunobiology, Yale School of Medicine, New Haven, Connecticut; 2 Department of Emergency Medicine, New York University Grossman School of Medicine, New York; 3 Division of General Internal Medicine, University of California, Davis, School of Medicine, Sacramento; 4 Section of General Internal Medicine, Department of Medicine, Yale School of Medicine, New Haven, Connecticut; 5 Department of Surgery, Yale School of Medicine, New Haven, Connecticut; 6 National Academies of Science, Engineering, and Medicine, Washington, DC; 7 Institute for Excellence in Health Equity, New York University Grossman School of Medicine, New York; 8 Department of Chronic Disease Epidemiology, Yale School of Public Health, New Haven, Connecticut; 9 Department of Surgery, Icahn School of Medicine at Mount Sinai, New York, New York

## Abstract

This cross-sectional study evaluates US medical school faculty diversity before and after introduction of the Liaison Committee on Medical Education’s diversity standards.

## Introduction

Diversity among medical school faculty is critical to provide support, belonging, and mentorship to all students, particularly those from marginalized backgrounds.^1,2^ In 2009, the Liaison Committee on Medical Education (LCME) introduced 2 diversity accreditation standards requiring every medical school to implement “policies and practices to achieve appropriate diversity among its students and faculty.”^3^ Implementation of these standards led to an increase in female, Black, Hispanic, and Asian medical students.^4^ However, beginning in 2025, the revised standards will not require medical schools to implement policies relating to faculty diversity. This study examines faculty diversity after introduction of the 2009 LCME diversity standards.

## Methods

We used deidentified data from the Association of American Medical Colleges (AAMC) faculty roster, which includes full-time faculty across schools of medicine and affiliated hospitals from 2002 to 2019. This study follows the Strengthening the Reporting of Observational Studies in Epidemiology (STROBE) reporting guideline for cross sectional studies and met criteria to be exempt from review and need for informed consent by the New York University Grossman School of Medicine’s institutional review board because data were deidentified. Sex, race, and ethnicity were self-reported and defined on the AAMC Faculty Roster website.^5^ Race and ethnicity included American Indian, Alaska Native, Native Hawaiian, and Pacific Islander; Asian; Black; Hispanic; and White.

We defined 3 critical periods for analysis: pre-LCME introduction (2002-2009), implementation (2009-2012), and post-LCME introduction (2012-2019). We calculated annual percentage change (APC) by gender, race, ethnicity, and their intersections, and compared pre-LCME and post-LCME periods using interrupted time series analysis. Statistical significance was defined as a 2-sided *P* < .05. Statistical analyses were performed using STATA version 18 (StataCorp). Data were analyzed from September to October 2024.

## Results

**Figure 1.**
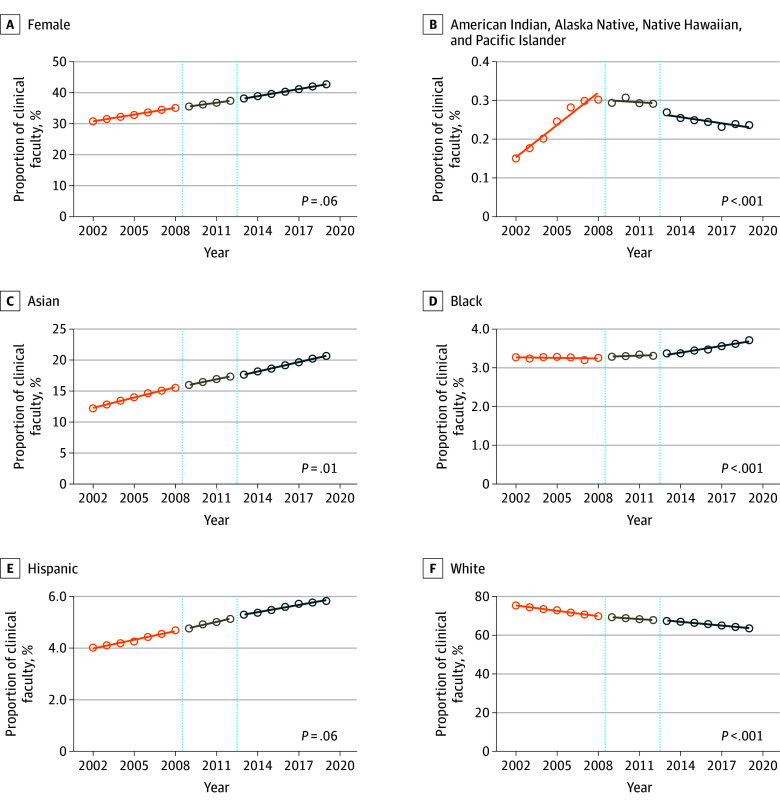
Faculty Representation by Sex, Race, and Ethnicity From 2002 to 2019 Annual faculty representation during 3 critical Liaison Committee on Medical Education (LCME) periods: pre-LCME diversity standards introduction from 2002 to 2009, implementation period from 2009 to 2012, and post-LCME diversity standards introduction from 2012 to 2019. *P*-values within graphs indicate significance from interrupted time series comparison between pre- and post-LCME diversity standards introduction periods (2002-2009 vs 2012-2019).

The total number of medical school faculty increased from 112 247 in 2002 to 187 838 in 2019. APC in female faculty did not change after the introduction of the 2009 LCME diversity standards (APC 0.72%; 95% CI, 0.68% to 0.76%; vs 0.76%; 95% CI, 0.73% to 0.79%; *P* = .06) (Figure 1A).

**Figure 2.**
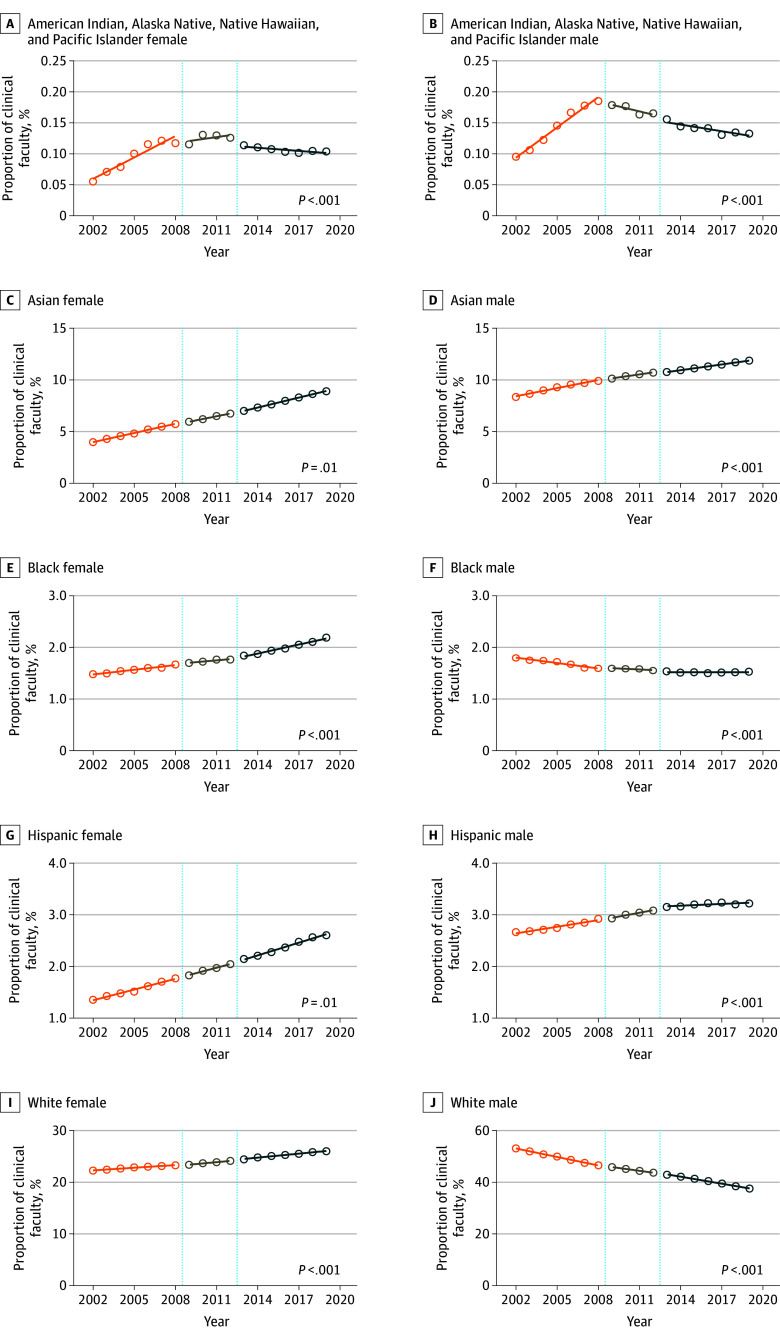
Faculty Representation by Intersection of Sex, Race, and Ethnicity From 2002 to 2019 Annual faculty representation by sex during 3 critical Liaison Committee on Medical Education (LCME) periods: pre-LCME diversity standards introduction from 2002 to 2009, implementation period from 2009 to 2012, and post-LCME diversity standards introduction from 2012 to 2019. *P*-values within graphs indicate significance from interrupted time series comparison between pre- and post-LCME diversity standards introduction periods (2002-2009 vs 2012-2019).

Among racial and ethnic groups, the decline in APC for White faculty significantly decreased after implementation (−0.92%; 95% CI, −0.98% to −0.86%; vs −0.64%; 95% CI, −0.72% to −0.57%; *P* < .001) (Figure 1B). APC for White female faculty increased after implementation (0.25%; 95% CI, 0.22% to 0.28% vs 0.17%; 95% CI, 0.14% to 0.19%; *P* < .001). The decline in APC for White male faculty decreased after implementation (−1.09%; 95% CI, −1.14% to −1.04% vs −0.90%; 95% CI, −0.96% to −0.85%; *P *< .001) (Figure 2). APC in overall Asian faculty significantly decreased after implementation, while there was an APC decrease for Asian male faculty, and an increase for Asian female faculty (Asian: APC, 0.55%; 95% CI, 0.51 to 0.60% vs 0.50%; 95% CI, 0.49% to 0.52%; *P* = .01; Asian male: APC, 0.26%; 95% CI, 0.22% to 0.30% vs 0.18%; 95% CI, 0.17% to 0.19%; *P* < .001; Asian female: APC, 0.29%; 95% CI, 0.28% to 0.31% vs 0.32%; 95% CI, 0.31% to 0.33%; *P* = .01) (Figure 1C, Figure 2).

LCME standards were associated with an APC increase for Black faculty (0.00%; 95% CI, −0.01% to 0.00%; vs 0.05%; 95% CI, 0.04% to 0.07%; *P* < .001) (Figure 1D). While APC in Black male faculty was decreasing before 2009, this decline halted after the LCME diversity standards were introduced (−0.03%; 95% CI, −0.04% to −0.02%; vs 0.00%; 95% CI, 0.00% to −0.01%; *P* < .001). After the standards were introduced, APC in Black female faculty slightly increased (0.03%; 95% CI, 0.02% to 0.04%; vs 0.05%; 95% CI, 0.05% to −0.06%; *P* < .001) (Figure 2).

While the LCME standards were not associated with APC difference for Hispanic faculty (0.11%; 95% CI, 0.09% to 0.13% vs 0.09%; 95% CI, 0.08% to 0.10%; *P* = .06)., APC increased for Hispanic female (0.06%; 95% CI, 0.05% to 0.08%; vs 0.08%; 95% CI, 0.07% to 0.09%; *P* = .03) and decreased for Hispanic male faculty (0.04%; 95% CI, 0.03% to 0.05%; vs 0.01%; 95% CI, 0.00% to 0.02%; *P* < .001) (Figure 2).

APC in American Indian, Alaska Native, Native Hawaiian, and Pacific Islander faculty significantly decreased after implementation (0.02%; 95% CI, 0.02% to 0.03%; vs −0.01%; 95% CI, −0.01% to 0.00%; *P* < .001) (Figure 1F). Similar associations were found for both female and male faculty within this group (female: 0.01%; 95% CI, 0.007% to 0.01% vs −0.001%; 95% CI, −0.002% to −0.003%; *P* < .001; male: 0.01%; 95% CI, 0.01% to 0.01% vs −0.003%; 95% CI, −0.005% to −0.001%; *P* < .001) (Figure 2).

## Discussion

Findings from this study suggest that while the 2009 LCME diversity standards were associated with gains in racial and ethnic diversity for medical students,^4^ the gains for faculty were not uniform. LCME diversity standards’ greater association with student diversity than faculty diversity may be because medical schools have more direct control over admissions policies, and potentially a more intentional focus on student diversity compared with faculty diversity, while faculty diversity may be constrained by slower hiring cycles and a more limited pool of underrepresented candidates. While representation of Black faculty increased with LCME standards introduction, the overall percentage of Black faculty remain small, and there was no gain for Black male faculty. The lack of Black male faculty in medicine limits role models for all students, particularly Black male students, making it difficult to mitigate the persistent lack of growth of Black men entering medicine. There is a persistent decline in Indigenous faculty, which highlights the need to promote Indigenous matriculants, graduates, and faculty.^6^ This study is limited by other potential factors that may influence faculty hiring, such as a rise in cluster hiring practices. However, with LCME removing faculty diversity requirements in 2025, future research is critical to assess its impact on hiring processes and faculty diversity.

## Notes

Supplement.
Data Sharing Statement
