# Supplementary material for: Medical School Faculty Diversity and the Liaison Committee on Medical Education’s Diversity Standards
Source: JAMA Netw Open. 2025 May 22;8(5):e2512096. doi: 10.1001/jamanetworkopen.2025.12096 (PMC12100449; doi:10.1001/jamanetworkopen.2025.12096)
Supplement: Supplement. — Data Sharing Statement [file jamanetwopen-e2512096-s001.pdf]

Data Sharing Statement

Nguyen. Medical School Faculty Diversity and the Liaison Committee on Medical Education's Diversity Standards. *JAMA Netw Open*. Published May 22, 2025.
doi:10.1001/jamanetworkopen.2025.12096

Data
Data available: No
